# Aortic Valve Neocuspidization with Glutaraldehyde-Treated Autologous Pericardium (Ozaki Procedure) - A Promising Surgical Technique

**DOI:** 10.21470/1678-9741-2019-0304

**Published:** 2019

**Authors:** Michel Pompeu Barros Oliveira Sá, Álvaro M. Perazzo, Konstantin Zhigalov, Roman Komarov, Bakytbek Kadyraliev, Soslan Enginoev, Jürgen Ennker, Aron Frederik Popov, Cesare Quarto, Alexander Weymann, Ricardo Carvalho Lima

**Affiliations:** 1 Division of Cardiovascular Surgery, Pronto-Socorro Cardiológico de Pernambuco - Prof. Luiz Tavares, PROCAPE, Recife, Brazil.; 2 University of Pernambuco, UPE, Recife, Brazil.; 3 Nucleus of Postgraduate and Research in Health Sciences of Faculty of Medical Sciences and Biological Sciences Institute, FCM/ICB, Recife, Brazil.; 4 Department of Thoracic and Cardiovascular Surgery, West German Heart and Vascular Center Essen, University Hospital of Essen, Germany; 5 Department of Cardiac Surgery, Sechenov First Moscow State Medical University, Moscow, Russia.; 6 Department of Cardiac Surgery, E.A. Vagner Perm State Medical University, S.G. Sukhanov Federal Center of Cardiovascular Surgery, Perm, Russia.; 7 Department of Cardiac Surgery, Federal Center for Cardiovascular Surgery, Russia; 8 Astrakhan State Medical University, Astrakhan, Russia.; 9 School of Medicine, Faculty of Health, University of Witten Herdecke, Witten, Germany.; 10 Department of Thoracic and Cardiovascular Surgery, University Medical Centre Tübingen, Tübingen, Germany.; 11 Department of Cardiothoracic Surgery, Royal Brompton & Harefield NHS Trust, London, UK.; 12 Department of Cardiac Surgery, European Medical School Oldenburg, Groningen, Carl von Ossietzky University Oldenburg, Oldenburg, Germany.

**Keywords:** Aortic Valve, Transcatheter Aortic Valve Replace, Pericardium, Heart Valve Prosthesis

## Abstract

In cases of aortic valve disease, prosthetic valves have been increasingly used for valve replacement, however, there are inherent problems with prostheses, and their quality in the so-called Third World countries is lower in comparison to new-generation models, which leads to shorter durability. Recently, transcatheter aortic valve replacement has been explored as a less invasive option for patients with high-risk surgical profile.

In this scenario, aortic valve neocuspidization (AVNeo) has emerged as another option, which can be applied to a wide spectrum of aortic valve diseases. Despite the promising results, this procedure is not widely spread among cardiac surgeons yet. Spurred on by the last publications, we went on to write an overview of the current practice of state-of-the-art AVNeo and its results.

**Table:** 

Abbreviations, acronyms & symbols	
AR	= Aortic regurgitation
AVNeo	= Aortic valve neocuspidization
AVR	= Aortic valve replacement
IE	= Infective endocarditis
SD	= Standard deviation

**Figure:**
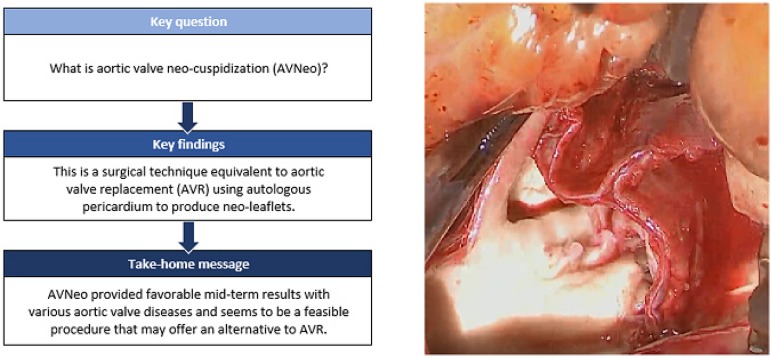

## INTRODUCTION

Ozaki et al.^[1]^ reported in 2011 their first institutional report about a surgical technique with which they replaced all three aortic valve leaflets using glutaraldehyde-treated autologous pericardium. Since this procedure is not widely spread among cardiac surgeons, but it has been gaining some ground, we aimed to assess the current practice of state-of-the-art aortic valve neocuspidization (AVNeo) and its results.

### Search Strategy

The search strategy using MEDLINE, from 1950 to June 2019, and the PubMed interface was: ("Aortic Valve"[Mesh] OR "Aortic Valve Stenosis"[Mesh] OR "Aortic Valve Prolapse"[Mesh] OR "Aortic Valve Insufficiency"[Mesh] OR "Bicuspid Aortic Valve" [Supplementary Concept] OR "Aortic Valve Disease" [Supplementary Concept] OR "Aortic Valve, Calcification of" [Supplementary Concept] OR "Heart Valve Diseases"[Mesh] OR "Heart Valve Prolapse"[Mesh] OR "Heart Valves"[Mesh]) AND ("Cardiac Valve Annuloplasty"[Mesh] OR "Transplantation, Autologous"[Mesh] OR "Pericardium"[Mesh] OR “Glutaraldehyde-Treated Autologous Pericardium” OR “aortic valve reconstruction” OR “neocuspidization” OR “Ozaki procedure” OR “Ozaki technique” OR “Ozaki method”).

### Search Outcome

Nine hundred and thirty-nine papers were found using the reported search on PubMed. From these, 11 papers were identified, which provided the best evidence to the topic.

## RESULTS

### Publications by Ozaki’s Group

**Fig. 1:**
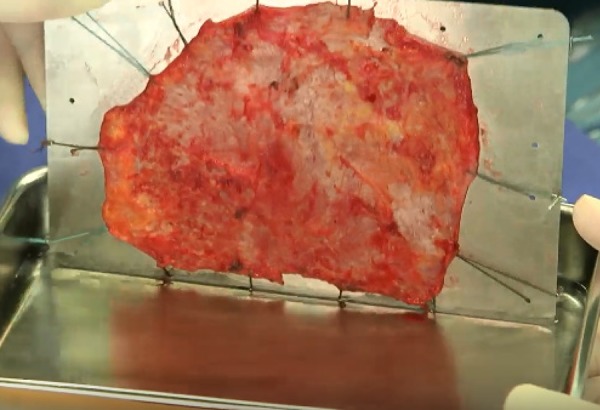
Glutaraldehyde-treated autologous pericardium.

**Fig. 2:**
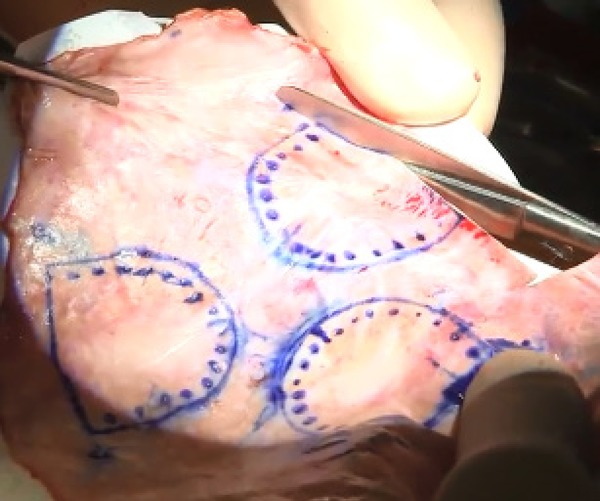
Trimming of treated autologous pericardium with the corresponding measured value using template.

In 2011, Ozaki et al.^[1]^ published their first case series with 88 patients from April 2007 to August 2009. They retrospectively reviewed these 88 cases and evaluated short-term and mid-term results. No operations converted to prosthetic valve replacement and no anticoagulation was administered postoperatively, except when the patients had atrial fibrillation. The preoperative mean gradient through the aortic valve was 81.6±31.1 mmHg and it decreased to 19.0±9.1 mmHg one week after surgery and to 12.9±5.8mmHg one year later. The degree of aortic regurgitation (AR) was always less than mild postoperatively. The first author describes that the preparation of the autologous pericardium was initiated by cleansing fat and other redundant tissues on the outer surface of the pericardium with the harmonic scalpel. Then, an excised pericardium (with a size of at least 7 x 8 cm) is treated with 0.6% glutaraldehyde solution for 10 minutes. The treated pericardium is rinsed for six minutes, three times, using physiological saline solution (Figure 1). Then, the pericardium is trimmed with the corresponding measured value using templates (Figure 2).

**Fig. 3:**
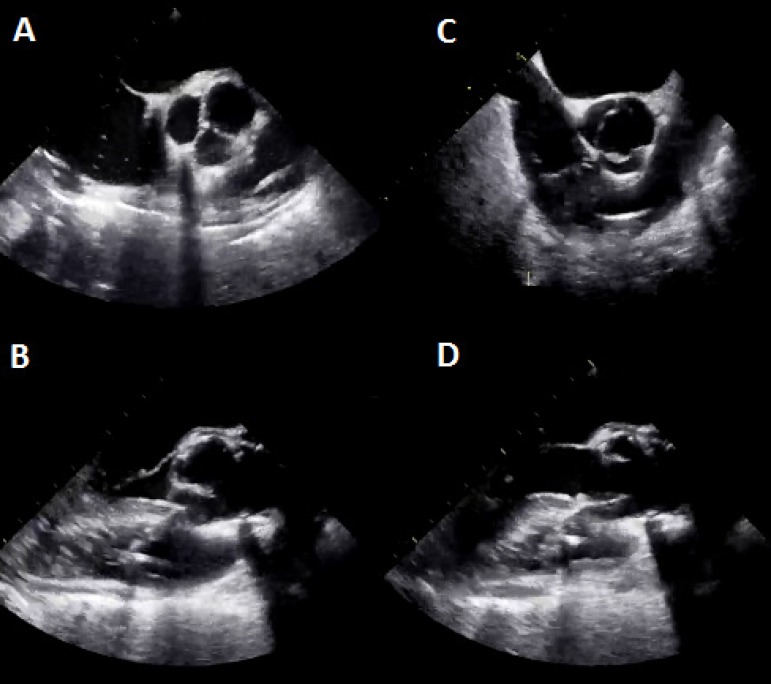
Echocardiographic pictures after the surgical procedure. A and B display adequate leaflet coaptation. C and D display the opening of the neo-aortic valve.

Ozaki et al.^[2]^ went on with their work and published a second paper with 404 cases of patients who underwent AVNeo, with a mean follow-up of 23.7±13.1 months. Survival rate was 87.7% at 53 months. Freedom from reoperation rate was 96.2%. Only two patients had to undergo reoperation, both because of infective endocarditis (IE). There were seven in-hospital mortalities resulting from a noncardiac cause. Postoperative echocardiography revealed good results with low peak pressure gradients after surgery (Figure 3).

In a study with a subset of 51 patients with age over 80 years, Ozaki et al.^[3]^ observed no conversion to AVR. Mean follow-up was 3.5 years. There were three in-hospital deaths due to noncardiac causes. No reoperation was needed. Survival at 56 months was 87.0%. No thromboembolic event occurred. Echocardiography 3.5 years after surgery revealed a low average peak pressure gradient and no moderate or severe AR was observed.

In 2013, a new publication of the same group came out^[4]^ showing a remarkable rate of freedom from reoperation of 96.7% at 73 months after surgery. Four reoperations were performed for IE. The other 412 patients had less than mild regurgitation. No thromboembolic events were observed.

**Fig. 4:**
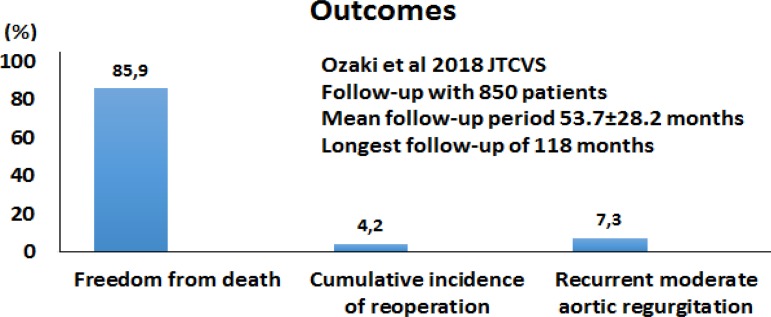
Results published by Ozaki et al.^[5]^.

In their last publication, in 2018^[5]^, 850 patients had already been operated on between April 2007 and December 2015 by Ozaki’s team. Preoperative echocardiography revealed a peak pressure gradient average of 68.9±36.3 mmHg with aortic stenosis that decreased to 19.5±10.3 mmHg one week after surgery and to 15.2±6.3 mmHg eight years after surgery. There was no conversion to aortic valve replacement (AVR). There were 16 in-hospital deaths. Fifteen patients needed reoperation (13 IE, one break of thread, and one tear of cusp case). See the results in Figure 4.

### Clinical Reports from Other Groups

Reuthebuch et al.^[6]^ operated on a total of 30 patients by means of the Ozaki technique between September 2015 and May 2017. The patients’ mean ± standard deviation (SD) age was 66.83±10.55 years, and 66.67% (n=20) of them were male. A combined aortic pathology of stenosis and regurgitation occurred in 11 patients (36.67%). Seven patients (23.33%) had pure aortic stenosis and 12 patients (40%) had regurgitation only. One patient (3.33%) had active endocarditis. Between postoperative day 30 and the three-month follow-up, one more patient died from aspiration pneumonia. No patient required reoperation or experienced an additional thromboembolic event within the first three months. One patient developed moderate aortic valve regurgitation postoperatively. This was found to be due to valvular endocarditis; the patient underwent reoperation five months after the initial operation and a biological valve was implanted. After three months, none of the patients had evidence of aortic valve stenosis. Moderate aortic valve regurgitation was seen in one patient (3.57%). Mild AR was seen in three patients (10.71%), whereas no or only trace AR was seen in the majority of patients (n=24; 85.71%). The mean transvalvular pressure gradient was 6.57±3.53 mmHg (n=22); and peak gradient was 13.51±8.88 mmHg.

Iida et al.^[7]^ performed AVNeo for aortic stenosis in 57 patients from December 2010 to June 2017. Their mean age was 77.5±8.8 years. Preoperative echocardiography revealed an average peak pressure gradient of 89±32.9 mmHg that decreased to 22±10.7 mmHg one week after the procedure and to 19.2±9.7 mmHg 20 months after the procedure. There were no conversions to AVR. There were two noncardiac-related deaths. Two patients underwent reoperation owing to IE and recurrent AR. The mean follow-up period was 30.4±20.8 months. Freedom from reoperation rates were 98.1% and 95.3% at 12 and 81 months of follow-up, respectively.

Nguyen et al.^[8]^ operated on nine patients with severe aortic valve diseases by means of an upper ministernotomy. The pericardium was harvested endoscopically, and then a ministernotomy was performed and the Ozaki procedure was accomplished in a similar manner to the conventional technique. No in-hospital or 30-day mortality was observed in their series, and no conversion to full sternotomy was required. Transthoracic echocardiography on discharge showed five competent valves and three valves with trivial regurgitation.

Mourad et al.^[9]^ carried out a prospective single-centre study including 52 consecutive patients who underwent AVNeo between September 2015 and March 2017 using autologous pericardium in 16 patients. Most patients presented with aortic stenosis or endocarditis. The mean age was 60±14 years. Early outcomes included one stroke, two patients needing short-term dialysis, and one death. During follow-up (mean 11.2±4.8 months), trace AR was observed in four patients; the mean pressure gradient was 6.8±2.9 mmHg. Three patients died later (of noncardiac reasons) and five patients needed reoperation due to endocarditis.

Iida et al.^[10]^ performed AVNeo for various aortic valve pathologies in 147 patients from December 2010 to October 2017. Of these patients, the aortic annulus dimensions were measured in 25 patients who underwent AVNeo for aortic valve disease as follow-up examination and they were compared with those measured in 15 patients who had normal aortic valves. No significant differences in the aortic annulus dimensions were observed between the patients who had undergone AVNeo and those who had normal aortic valves. The authors concluded that the movement of the aortic annulus after AVNeo is comparable with that of the aortic annulus of a normal aortic valve, and thus AVNeo can be regarded as a more physiological operation in that it maintains the characteristics of the aortic valve similar to those of a normal aortic valve (which, in turn, does not happen when patients undergo AVR).

Krane et al.^[11]^ operated on 77 patients undergoing AVNeo following the Osaki procedure between October 2016 and August 2018. Mean age was 54.9±16.5 years, and aortic stenosis was present in 84.4% and insufficiency in 15.6% of the patients. At 1.76-year follow-up, freedom from reoperation was 97.4%. Two patients (2.6%) presented with moderate to severe aortic insufficiency after the procedure. Both received a prosthetic AVR during the same hospital stay. At discharge, mean pressure gradient was 9.3±4.2 mmHg, which decreased to a mean aortic gradient of 1.6±3.4 mmHg at six to 12 months. The authors concluded that AVNeo following the Ozaki procedure revealed excellent early hemodynamic results in terms of effective orifice area, pressure gradients, and prosthesis-patient mismatch.

### Every Like Is Not the Same, But One May Give an Idea of the Other

Among the various repair techniques for aortic valve diseases, the leaflet extension technique for AR has been used in cases of severe cusp retraction that cannot be corrected by means of other techniques^[12-14]^. The ideal material for leaflet extension remains controversial and one of them is also the autologous pericardium advocated by Kwak et al.^[12]^, who evaluated the 20-year clinical outcomes of the leaflet extension technique for AR caused by rheumatic valvular disease. We would like to highlight that this is not the same technique as Ozaki’s, since the leaflets are not resected, but the fact that the autologous pericardium is also employed might well give us an idea of the durability of this material.

In that study^[12]^, there were no cases of operative mortality, but postoperative complications occurred in five patients. Overall survival at 10 and 20 years was 93.5% and 87.1%, respectively. Freedom from reoperation at 10 and 20 years was 96.7% and 66.6%, respectively. Kwak et al.^[12]^ concluded that long-term results of the leaflet extension technique showed acceptable durability.

### Clinical Bottom Line

Current literature suggests that AVNeo with Ozaki procedure seems to be a promising technique and good alternative to AVR with prostheses. Long-term results with a longer follow-up of 10 to 15 years are to be published yet. The authors who published their first experiences with this technique achieved comparable clinical outcomes to conventional therapies with good hemodynamics and a warfarin-free condition. Nevertheless, clinical trials are still necessary.

**Table:** 

Authors' roles & responsibilities	
MPBOS	Substantial contributions to the conception or design of the work; or the acquisition, analysis, or interpretation of data for the work; drafting the work or revising it critically for important intellectual content; agreement to be accountable for all aspects of the work in ensuring that questions related to the accuracy or integrity of any part of the work are appropriately investigated and resolved; final approval of the version to be published
AMP	Substantial contributions to the conception or design of the work; or the acquisition, analysis, or interpretation of data for the work; drafting the work or revising it critically for important intellectual content; final approval of the version to be published
KZ	Substantial contributions to the conception or design of the work; revising it critically for important intellectual content; final approval of the version to be published
RK	Substantial contributions to the conception or design of the work; revising it critically for important intellectual content; final approval of the version to be published
BK	Substantial contributions to the conception or design of the work; revising it critically for important intellectual content; final approval of the version to be published
SE	Substantial contributions to the conception or design of the work; revising it critically for important intellectual content; final approval of the version to be published
JE	Substantial contributions to the conception or design of the work; revising it critically for important intellectual content; final approval of the version to be published
AFP	Substantial contributions to the conception or design of the work; revising it critically for important intellectual content; final approval of the version to be published
CQ	Substantial contributions to the conception or design of the work; revising it critically for important intellectual content; final approval of the version to be published
AW	Substantial contributions to the conception or design of the work; revising it critically for important intellectual content; final approval of the version to be published
RCL	Substantial contributions to the conception or design of the work; revising it critically for important intellectual content; final approval of the version to be published
